# A National Study on Training Innovation in US Medical Education

**DOI:** 10.7759/cureus.46433

**Published:** 2023-10-03

**Authors:** David I Hindin, Michael Mazzei, Shreyas Chandragiri, Lauren DuBose, Dominick Threeton, Jerry Lassa, Dan E Azagury

**Affiliations:** 1 Department of Surgery, Stanford University School of Medicine, Stanford, USA; 2 Department of Surgery, Lewis Katz School of Medicine, Temple University, Philadelphia, USA; 3 Department of Surgery, Sidney Kimmel Medical College, Philadelphia, USA; 4 Department of Bioengineering, Temple University, Philadelphia, USA; 5 Department of Mathematics, Northwestern University, Evanston, USA

**Keywords:** narrative medicine, medical humanities, design thinking, technology transfer, enrepreneurship, innovation in medical education, innovation in medical teaching, innovation, medical education

## Abstract

Introduction

Traditional medical education has leaned heavily on memorization, pattern recognition, and learned algorithmic thinking. Increasingly, however, creativity and innovation are becoming recognized as a valuable component of medical education. In this national survey of Association of American Medical Colleges (AAMC) member institutions, we seek to examine the current landscape of exposure to innovation-related training within the formal academic setting.

Methods

Surveys were distributed to 168 of 171 AAMC-member institutions (the remaining three were excluded from the study for lack of publicly available contact information). Questions assessed exposure for medical students among four defined innovation pillars as follows: (1) medical humanities, (2) design thinking, (3) entrepreneurship, or (4) technology transfer. Chi-squared analysis was used to assess statistical significance between schools, comparing schools ranked in the top 20 by the US News and World Report against non-top 20 respondents, and comparing schools that serve as National Institutes of Health (NIH) Clinical and Translational Science Awards (CTSA) program hubs against non-CTSA schools. Heat maps for geospatial visualization of data were created using ArcGIS (ArcMAP 10.6) software (Redlands, CA: Environmental Systems Research Institute).

Results

The overall response rate was 94.2% with 161 schools responding. Among respondents, 101 (63%) reported having medical humanities curricula at their institution. Design thinking offerings were noted at 51/161 (32%) institutions. Support for entrepreneurship was observed at 51/161 institutions (32%), and technology transfer infrastructure was confirmed at 42/161 (26%) of institutions. No statistically significant difference was found between top 20 schools and lower 141 schools when comparing schools with no innovation programs or one or more innovation programs (p=0.592), or all four innovation programs (p=0.108). CTSA programs, however, did show a statistically significant difference (p<0.00001) when comparing schools with no innovation programs vs. one or more programs, but not when comparing to schools with all four innovation programs (p=0.639).

Conclusion

This study demonstrated an overwhelming prevalence of innovation programs in today’s AAMC medical schools, with over 75% of surveyed institutions offering at least one innovation program. No statistically significant trend was seen in the presence of zero programs, one or more, or all four programs between top 20 programs and the remaining 141. CTSA hub schools, however, were significantly more likely to have at least one program vs. none compared to non-CTSA hub schools. Future studies would be valuable to assess the long-term impact of this trend on medical student education.

## Introduction

Each fall, thousands of first-year medical students across the United States begin their journey into the world of medicine. From the outset, the task at hand for these students is clear - to memorize as much information as possible, a process ruefully described by generations of upperclassmen as akin to "sipping water from a firehose." Indeed, as far back as the early 1900s, when the Flexner report helped distill medical education into discrete scientific curricula, medical school training has long prioritized memorizing facts and algorithmic thinking over utilizing creativity and imagination [1,2].

Recently, however, a growing number of medical schools have begun to place an emphasis on encouraging creativity and innovation among their students within a variety of academic contexts [3-6]. These changes range from optional extracurricular experiences to mandatory courses interwoven with the overall didactic curriculum.

At some institutions, professors of fine art guide medical students to critically observe various paintings [7]. The process known as visual literacy training is designed to improve a medical student’s observational and physical examination skills. At other centers, students are ushered through a curriculum that includes topics such as reading fictional literature, studying anthropologic texts, and even producing a written narrative of their own experiences, all designed to help budding physicians grow more attuned to the humanistic elements of medicine [8]. Meanwhile, other schools have begun to introduce a process called design thinking into their curricula, equipping students with skills that can be leveraged to develop creative, innovative solutions to problems their patients may face.

The trend is not just limited to the United States, either. Recent publications from the United Kingdom signal not only growing support for creative writing within medicine but also the possibility of placing a greater emphasis on seeking out medical students based on traits that predict one’s creative potential [9-11].

While these examples each point to a burgeoning emphasis on creativity and innovation within medical school, research into this growing trend remains sparse. This national survey sought to examine the ways in which creativity and imagination are beginning to emerge as a focus within medical schools.

## Materials and methods

A national survey was sent electronically to individual deans of academic affairs at 168 Association of American Medical Colleges (AAMC)-member medical schools. Three additional programs (171 total programs) did not have a website or publicly available contact information and were excluded from the study. Questions were designed to query whether the school provided exposure for medical students in each of the following four distinct pillars: (1) medical humanities, (2) design thinking, (3) entrepreneurship, or (4) technology transfer. These categories were selected to allow for the broadest possible representation of creativity and innovation within the medical field. Non-responders received two separate follow-up emails. If no response was received following these reminder emails, individual academic offices were contacted via a telephone call in order to fill out the survey manually over the phone. If an academic officer could not be reached by telephone, the survey was manually filled out based on publicly available information on the school’s website (a series of search words were used when querying each institution’s website. The study was deemed exempt from review by Temple University Hospital and Stanford University’s Institutional Review Board.

Survey responses were examined to assess overall percentages among each of the four categories. In order to provide geospatial visualization of data, all results were then correlated to a heat map using ArcGIS (ArcMAP 10.6) software (Redlands, CA: Environmental Systems Research Institute). For each medical school, survey responses were plotted on the map in correlation to that school’s zip code.

To examine the potential differences between research-intensive and primary care-oriented institutions, the US News and World Report rankings were used to compare the institutional rank of each of these categories [12]. Pearson correlation coefficients were calculated to compare responses based on research-ranked order of institutions, in comparison to primary care-ranked order. As an alternative approach to assessing for differences in responses based on institutional research emphasis, responses were also examined with respect to each institution’s NIH Clinical and Translational Science Awards (CTSA) program status.

The four pillars, described in further detail below, were selected in order to broadly categorize individual examples of curriculum-based exposure to creativity and innovation and to more easily delineate overall trends.

Medical humanities

The overall category of topics within medical humanities is both broad and interdisciplinary. Depending on the context, the category of medical humanities is used here to refer to the intersection of medicine with more traditional humanities (literature, philosophy, ethics, religion, history, social sciences, anthropology, cultural studies, psychology, sociology, health geography), or art-centric topics (literature, theater, film), with a focus on their application to the field of medicine and medical education [13]. This category also encompasses narrative medicine and similar creative writing programs, in which a component of the curriculum involves reflective, experience-based writing from the student.

Design thinking

Alternately referred to as health design, design thinking, and biodesign, this pillar refers, in particular, to students who are trained in a reproducible process of innovation. The category includes programs in which students are taught skills such as needs finding, concept generation, and strategy development. This category also applies to institutions in which students are provided with physical equipment and tools to develop prototypes and test these in a real population. Frequently, design thinking programs are broadly collaborative, involving teamwork and input from across multiple disciplines and departments.

Entrepreneurship

Survey responses that touched on a variety of business and entrepreneurial contexts were included in this category. This pillar includes experiences ranging from school-sponsored hackathons for medical students interested in developing a new health technology startup to an academic curriculum focused on exposing medical students to business-oriented classes. As with design thinking programs, entrepreneurship curricula and programs tended to span multiple departments and schools within a given institution.

Technology transfer

Broadly speaking, the concept of technology transfer in this context encompasses the so-called “bench to bedside” process of transitioning an institution’s intellectual property into a marketable product. This pillar was designed to capture a broad range of potential responses as a proxy for infrastructure with the capability of supporting innovation, ranging from direct student involvement with an institution’s technology transfer department (often in due diligence and market assessment research) to active participation in the basic science process of developing a marketable technology.

Data from the survey responses was also assessed for statistical significance using chi-squared analysis. Responses of schools with all four programs, schools with zero programs, and schools with one or more programs were examined. Schools ranked by the US News and World Report as top 20 medical schools were compared with the remaining 141 schools. Similarly, results from schools that were classified as CTSA hubs during the year of the survey were compared with schools that were not.

## Results

In total, data were obtained for 161 of 171 medical schools surveyed, for an overall response rate of 94.2%. Among all schools, 101 of 161 (63%) reported having medical humanities exposure for medical students at their institution. Design thinking programs or resources were noted at 51 of 161 (32%) institutions. Resources or programs for entrepreneurship were observed at 51 of 161 (32%) institutions, and technology transfer infrastructure was confirmed at 42 of 161 (26%) institutions (Table 1).

**Table 1:** Overview of medical schools surveyed and total responses per category.

Total number of schools surveyed overall	161
Total number of schools with a program for medical humanities	101
Total number of schools with a program for design thinking	51
Total number of schools with a program for entrepreneurship	51
Total number of schools with a program for technology transfer	42
Total number of schools with a program for both medical humanities and entrepreneurship	41
Total number of schools with a program for both medical humanities and technology transfer	33
Total number of schools with a program for both medical humanities and design thinking	40
Total number of schools with a program for both design thinking and entrepreneurship	28
Total number of schools with a program for both design thinking and technology transfer	23
Total number of schools with a program for both entrepreneurship and technology transfer	27
Total number of schools with 1 or more of the above programs	121
Total number of schools with 2 or more of the above programs	72
Total number of schools with 3 or more of the above programs	36
Total number of schools with all 4 of the above programs	16

To examine the potential correlation between the US News and World Report rankings and survey responses, we compared respondents in two distinct categories as follows: one group with institutions organized by their research ranking and one group with institutions ranked based on primary care. Pearson correlation coefficients were calculated, comparing the ordinal variables within research ranking and primary care ranking to survey responses. The resulting R values were -0.0055 and 0.0528, respectively, indicating a lack of correlation between survey responses and the corresponding institutional rank, whether as a research institution or primary care institution.

Survey responses were further compared with the top 20 research institution rankings from the US News and World Report list to assess for potential correlation between these top-ranked institutions and the presence or absence of the programs discussed. Among the top 20 schools, 16 (80%) had one or more of the surveyed programs, compared with the general survey pool of 110 among the remaining 141 (approx. 78%) schools surveyed (Table 2). Chi-squared analysis showed that this was not a statistically significant difference, with a p-value of 0.592. Similarly, there was no statistically significant difference between the proportion of top-20 schools with all four innovation programs (four schools or 20%) compared with the remaining schools (13 of 141 schools or 9.2%), with a p-value of 0.108. There was also no statistically significant difference between top 20 schools with zero innovation programs (four schools or 20%) compared with the remaining schools (36 or 25.5%), with a p-value of 0.592. 

**Table 2:** Quantity of innovation programs by medical school rank.

Variables	Top 20 ranked school	Lower 141 ranked school	p-Value
Total number of schools with all 4 innovation programs	4	12	0.108
Total number of schools with 1 or more innovation programs	16	105	0.592
Total number of schools with 0 innovation programs	4	36	0.592

When sorting results based on CTSA status, 40 respondents of CTSA hub programs (77%) had one or more of the surveyed innovation programs, in comparison with 41 non-CTSA hub programs (37.6%). The chi-squared analysis demonstrated this to be a statistically significant difference, with a p<0.00001 (Table 3). However, no statistically significant difference was found when comparing CTSA hub schools with all four programs compared with non-hub schools (p=0.639).

**Table 3:** Quantity of innovation programs by CTSA hub status. CTSA: Clinical and Translational Science Awards

Variables	CTSA hub school	Non-CTSA hub school	p-Value
Total number of schools with all 4 innovation programs	6	10	0.639
Total number of schools with 1-3 innovation programs	40	28	<0.00001
Total number of schools with 0 innovation programs	12	81	<0.00001

Interestingly, a statistically significant correlation was seen between a school’s CTSA hub status and whether it was also ranked in the US News and World Report's top 20; 17 of the top 20 schools (85%) were CTSA hubs, whereas only 35 of the remaining schools (25%) were CTSA hubs (p<0.00001).

We also examined each individual type of innovation program, comparing these separately with the school’s US News and World Rank status and with whether the school was a CTSA hub (Tables 4, 5). Among all cases, the only statistically significant relationship was seen among the top 20 ranked schools with respect to technology transfer programs; 50% of these schools had a program in technology transfer, while only 22.7% of non-top 20 ranked schools had a similar program (p=0.009) (Table 4).

**Table 4:** Types of innovation programs by medical school rank.

Variables	Top 20 ranked school	Lower 141 ranked school	p-Value
Medical humanities program	15	86	0.225
Design thinking program	10	41	0.060
Entrepreneurship program	10	41	0.060
Technology transfer program	10	32	0.009

**Table 5:** Types of innovation programs by CTSA hub status. CTSA: Clinical and Translational Science Awards

Variables	CTSA hub school	Non-CTSA hub school	p-Value
Medical humanities program	35	66	0.407
Design thinking program	20	31	0.201
Entrepreneurship program	18	33	0.306
Technology transfer program	17	25	0.187

**Figure 1:**
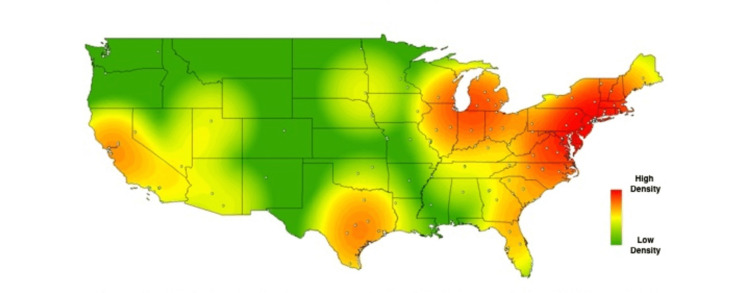
Medical school innovation design thinking respondents. Medical schools who answered "yes" to having a design thinking-related program available to medical students.

**Figure 2:**
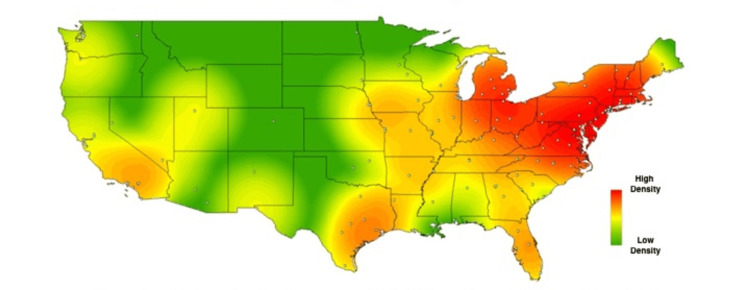
Medical school innovation entrepreneurship. Medical schools who answered "yes" to offering entrepreneurship-related resources to medical students.

**Figure 3:**
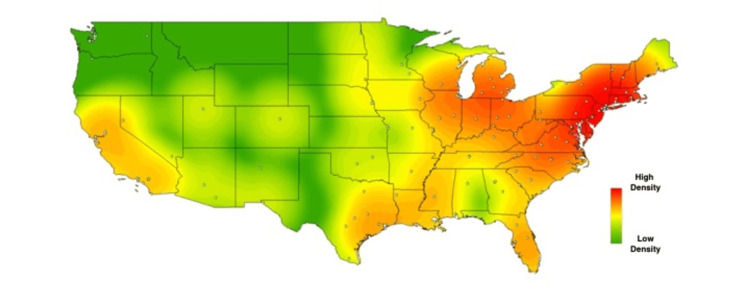
Medical school innovation medical humanities. Medical schools who answered "yes" to providing humanities-related programs for medical students.

**Figure 4:**
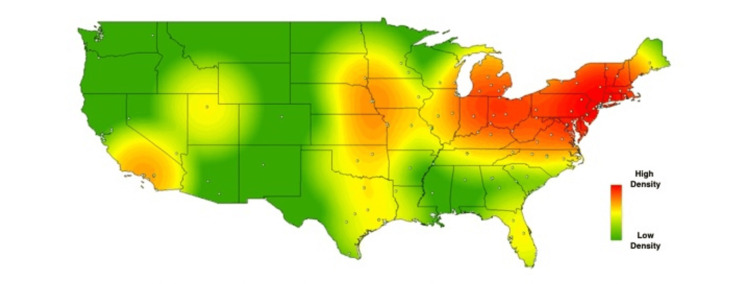
Medical school innovation technology transfer. Medical schools who answered "yes" to providing technology transfer programs for medical students.

**Figure 5:**
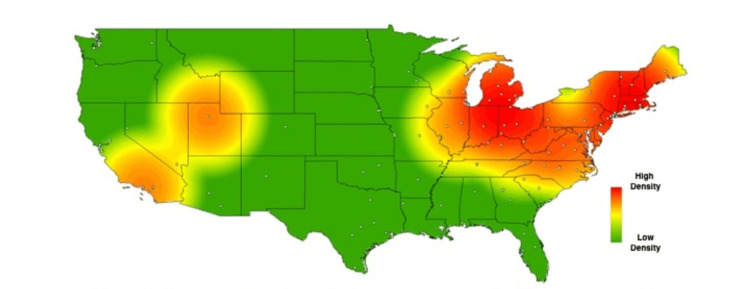
Medical school innovation "all four" respondents. Responses of medical schools who answered "yes" to the presence of all four programs surveyed.

**Figure 6:**
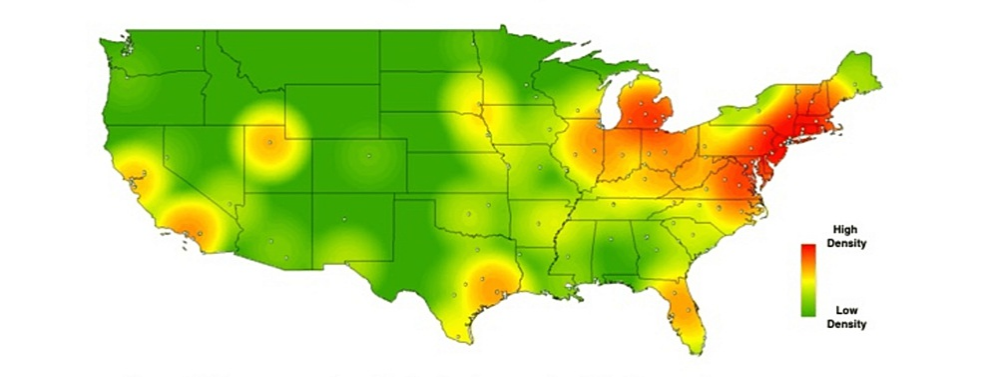
Medical school innovation weighted responses. Responses of medical schools answering "yes" for a given program were weighted using a squared weighting system, based on the total amount of innovation programs (e.g., schools with one innovation program provide a point value of 1^2^ {or 1}, schools with 2 innovation programs provide a point value of 2^2^ {or 4}, etc.).

In order to visually contextualize these survey responses, heat maps were created for the above results, using each school’s zip code to geospatially plot its location on the map. Each school’s location is represented on the maps by a single, white-colored dot. As described in methods, commercial heat-mapping software (ArcGIS ArcMAP 10.6) was then used to apply a halo of color for locations on the map responding “yes,” to a given question, with increasing intensity correlating to the amount of “yes” responses in a particular region (Figures 1-6). These heat maps provide a visual illustration to highlight the differences between the geographic density of schools overall and the frequency of specific programs within these schools.

Individual maps were created to highlight each of the surveyed pillars (entrepreneurship, medical humanities, etc.), as well as a series of additional maps created to show the distribution of responses answering “yes” to the presence of all four programs. One such map is shown in Figure 6, in which institutions with sequentially more innovation-geared programs are weighted more heavily - each program’s total number of innovation programs is squared. Programs with one innovation factor provide a point value of 1^2^ {or 1}, programs with two innovation factors provide a point value of 2^2^ {or 4}, programs with three innovation factors provide a point value of 3^2^ {or 9}, and programs with four innovation factors provide a point value of 4^2^ {or 16}, for the heat map.

While these findings are by nature qualitative, a few observations can be made. Among types of innovation programs, the program represented most frequently at institutions was medical humanities (63%), with technology transfer being represented least frequently 26%). Overall, the highest density of institutions with innovation-geared programs was seen in the Northeastern United States, with the second-largest focus of these programs in California. The lowest density of innovation programs was seen in the Midwest.

## Discussion

The data collected in this survey points clearly towards an emphasis on AAMC-based medical education in which a priority is being placed on skills relating to creativity and innovation for medical students. The reasons for this trend are likely multifactorial. For some institutions, encouraging students to be more creative is part of an overall effort to preserve the humanistic skills that allow individuals to be insightful, empathetic doctors:

How did the student interpret a patient’s narrative, what issues, dilemmas, or questions (personal as well as professional) were brought up during a consultation? … It seems to open up the reflective space, for example, by stepping into another’s shoes and attempting to view the world from another’s position [10].

From a more pragmatic perspective, training doctors to be more creative may be equally critical to the field of medicine in the United States as it is for producing well-rounded physicians. Some sources, for instance, point toward a 2010 report from the National Academies of Science, which found America’s markers for long-term sources of innovation to be falling behind much of the developed world [10]. Training doctors to be more effective innovators would therefore represent a long-term investment into our national intellectual capital for driving innovation.

This national survey sought to produce a snapshot as accurate as possible into the state of innovation and creativity exposure within medical school. Nonetheless, it is possible that the above results are limited by the constraints of our study design. Furthermore, due to the nature of our study, this snapshot is able to demonstrate current programs within medical schools but does not show the overall changes over time. To gain deeper insight into these trends, we plan to repeat our survey in the future. In addition, future surveys may help us determine whether there is a tendency for schools which add one of these programs to additional programs in a follow-on fashion [13].

While appropriate academic officials were selected to be contacted for completion of the survey, it is certainly possible that at larger medical institutions, even these academic officials may not have been aware of smaller, or newer programs being developed that were available to medical students, especially in the technology transfer and entrepreneurship sectors [14].

Similarly, for institutions in which a suitable academic official could not be contacted for survey completion via email or telephone, it is possible that the available public website information listed for academic programs was incomplete. In all of these scenarios, results would have been skewed towards the inclusion of fewer creativity/innovation programs for students, rather than more [14].

The authors recognize that neither a school’s US News and World Report Ranking nor its CTSA hub status can serve as a definitive indicator of the institution’s research goals and emphasis. The US News and World Rankings themselves are a hybrid of multiple factors extending well beyond an institution’s research activity and include factors such as median Medical College Admission Test (MCAT) score, school acceptance rates, and undergraduate Grade Point Average (GPA) of matriculating students. Meanwhile, the NIH Clinical and Translational Science Awards (CTSA) Program is geared towards a specific subset of research that may not accurately demonstrate the full breadth of an academic institution’s laboratory pursuits [15].

As discussed above in the Results section, we did not find a strong correlation between a school’s US News and World Report ranking and the presence of academic programs that nurture innovation. While there was a slightly increased proportion among the top 20-ranked schools, this was not found to be statistically significant; the programs were found in large numbers across non-top 20 institutions, as well. Among top 20 schools with innovation programs, we did see a statistically significant propensity towards the likelihood of having a technology transfer program (50% of top 20 ranked programs as opposed to lower 141 ranked), but it is not clear whether this has practical implications [15].

In contrast, a statistically significant correlation was seen between a school’s CTSA status and its emphasis on innovation programs. Interestingly, while top 20 status was not independently correlated with innovation emphasis, we did find this ranking to be strongly correlated with whether or not a school was a CTSA hub, suggesting that these two categories, when both present, may be independently predictive of innovation emphasis [16,17].

While the heat maps produced from our study did not point towards a single overriding trend, we did note a bicoastal pattern of greatest intensity in nearly all of our maps. Notably, while the areas of greatest intensity do share some correlation with the distribution of schools, a visual assessment of the images immediately suggests trends that extend beyond simple differences in population density. Interestingly, the areas we noted of greatest intensity correlate to previous studies demonstrating the geographic density of patents filed per square kilometer [18], as well as areas of the United States with the greatest amounts of venture capital investments [19].

Limitations

As outlined above, this study relied in part on data from interviews with academic officials who may potentially be unaware of newer programs at their own institutions. Similarly, website data may also be potentially outdated or incomplete. Finally, both the US News and World Report Rankings and CTSA hub statuses are unable to completely capture the full nuances of an institution's offerings in innovation and creativity.

## Conclusions

This study was able to capture creativity curriculum data on nearly all of the US medical schools surveyed. Taken as a whole, the majority of schools surveyed overall offered at least one innovation program.

While a range of rationales may help explain the increasing support for nurturing skills for creativity and innovation among medical students, the long-term impact of this new trend has yet to be seen. To better assess the value of this new trend, it will be necessary to determine suitable endpoints and measure longitudinal data among medical students exposed to these new academic trends. One thing, however, is clear - medical schools across the United States are committed to training our next generation of doctors to be innovative and creative.
